# Immunophenotypic Characterization of Tumor-Associated Macrophages in Oral Carcinogenesis: An Observational Study

**DOI:** 10.3390/ijms27156889

**Published:** 2026-08-01

**Authors:** Mafalda Alves, Sara Ferreira, Fernanda Garcês, Carolina Espíndula, Filomena Salazar, José Júlio Pacheco, Márcio Diniz-Freitas, Carlos Lopes, Saman Warnakulasuriya, Leonor Delgado, Luis Monteiro

**Affiliations:** 1 UNIPRO-Oral Pathology and Rehabilitation Research Unit, IUCS-CESPU, University Institute of Health Sciences, 4585-116 Gandra, Portugal; mafaldaalvesjp@gmail.com (M.A.);; 2 Medical-Surgical Dentistry Research Group, Health Research Institute of Santiago de Compostela, School of Medicine and Dentistry, Santiago de Compostela University, 15703 Santiago de Compostela, Spain; 3 The WHO Collaborating Centre for Oral Cancer, King’s College London, London SE1 9RT, UK

**Keywords:** oral squamous cell carcinoma, tumor-associated macrophages, macrophage polarization, CD68, CD163, CD80, oral carcinogenesis, tumor microenvironment, immunohistochemistry

## Abstract

Tumor-associated macrophages are key components of the tumor microenvironment in oral carcinogenesis; however, their role is not yet fully understood. We aimed to characterize the expression of macrophage (M) markers across the spectrum of oral carcinogenesis, including oral leukoplakias (OL) and oral squamous cell carcinomas (OSCCs). This immunohistochemical study analyzed the macrophage density (cells/mm^2^) based on the expression of CD68 (pan-macrophage), CD163 (M2), and CD80 (M1) in 81 samples, including healthy mucosa (*n* = 16), OL (*n* = 30), and OSCC (*n* = 35), in both epithelial and subepithelial compartments. All markers showed a progressive increase across the study groups, with significantly higher densities in OSCC than in healthy mucosa (*p* < 0.001), consistently higher in the subepithelial compartment. CD163 was the most expressed marker (increasing from 78.96 cells/mm^2^ in healthy mucosa to 300.56 cells/mm^2^ in OSCC), whereas CD80 was the least abundant, particularly in OSCC (35.66 cells/mm^2^ in healthy tissue to 188.49 cells/mm^2^ in OSCC). No significant associations were observed between biomarker expression and clinicopathological variables. These results support the involvement of macrophages during oral carcinogenesis, particularly CD163+ (M2) cells, and highlight the potential prognostic and therapeutic relevance of these markers.

## 1. Introduction

Oral cancer is a public health problem with a high incidence worldwide, remaining one of the most important causes of death in developed countries [1,2]. Despite advances in diagnostic and therapeutic strategies, the five-year survival rate for patients with oral cavity cancer remains approximately 50% [2]. Tobacco use and excessive alcohol consumption, as well as infection with high-risk Human papillomavirus (HPV) are considered the major risk factors for oral cancer although patient’s socioeconomic status and patient’s lifestyle have been associated with oral cancer [3,4]. Late-stage tumor presentation also significantly influences the prognosis of the disease [5,6]. Oral squamous cell carcinoma (OSCC) is the most common form of oral cancer and accounts for approximately 90% of the oral cancers [7,8].

Current evidence indicates that oral carcinogenesis occurs in stages, manifesting clinically at an early stage through the appearance of oral potentially malignant disorders (OPMDs), which may progress to malignancy [9,10]. OPMDs include conditions such as oral leukoplakia, erythroplakia, oral lichen planus and, lichenoid lesions, oral submucosal fibrosis and proliferative verrucous leukoplakia, the latter having the highest rate of malignant transformation [11].

Tumors are not simply a collection of proliferating malignant cells, but rather complex systems composed of different cell types that form the tumor microenvironment (TME), establishing an interactive network that contributes to tumor progression [8,9]. The TME can exert both tumor-promoting and tumor-suppressive effects, influenced by factors such as hypoxia, pH, angiogenesis, and metabolism [12,13,14].

Macrophages, as important cells of the immune system, play a central role in carcinogenesis and tumor progression. They can be classified into the following two main subtypes: M1, associated with classical activation, and M2, associated with alternative activation [15,16]. Unlike macrophages in healthy tissues, tumor-associated macrophages (TAMs) may exhibit deviant functions such as phagocytosis and antigen presentation, thereby promoting tumor progression [17]. They are among the most influential cells in tumor progression, and their presence is associated with a poorer prognosis [16]. The M1 phenotype is associated with host defense against infections and antitumor activity, while the M2 phenotype is involved in tissue repair and the promotion of immune tolerance [18]. Various therapeutic approaches aimed at modulating the activity and signaling mechanisms of macrophages have been explored in cancer research. Nevertheless, further research is needed to deepen our understanding of the role of TAMs in head and neck cancer and improve the development of new therapeutic strategies [19].

Although there is growing interest in the role of the tumor microenvironment in OSCC, the relationship between macrophage infiltration and polarization remains poorly understood [1]. A combined analysis of markers associated with the total macrophage population and the M1/M2 phenotypes may provide a deeper understanding of the tumor immune profile and its potential associations with clinicopathological and prognostic parameters [5]. The aim of this study was to evaluate the expression of CD68, CD163 (M2), and CD80 (M1) in oral leukoplakias (OL), oral squamous cell carcinoma (OSCC) samples, and healthy oral mucosa, analyzing their expression, distribution, and association with the clinicopathological characteristics of the patients.

## 2. Results

### 2.1. Demographic and Clinical Characteristics

A total of 81 cases were analyzed, divided into the following four diagnostic groups: 16 with normal oral mucosa, 14 with epithelial hyperplasia, 16 with epithelial dysplasia, and 35 with OSCC. The overall median age was 62.0 years (IQR, 50.0–75.5), with no significant difference among the groups (*p* = 0.226). Males predominated in the overall sample (54/81, 66.7%) and in most groups although the difference was not statistically significant (*p* = 0.344) (Table 1).

Regarding anatomical location, the tongue was the most common site in the overall sample (28/81, 34.6%), followed by the buccal mucosa and the alveolar ridge (12/81 each, 14.8%), the lip and floor of the mouth with eight (9.9%) cases each, the retromolar trigone (six; 7.4%), the palate (four; 4.9%) and three cases in mouth not otherwise specified (NOS) (3.7%). Lesions of the tongue and floor of the mouth accounted for 23.5% of all cases and were comparatively more frequent among OSCC (34.3%), while all samples of normal mucosa were located elsewhere; the overall difference between groups did not reach statistical significance (*p* = 0.055).

The demographic and clinical characteristics of the study population and other variables including histological grading for OL and OSCC are summarized in Table 1.

### 2.2. Macrophage Density Across Diagnostic Groups

**Figure 1:**
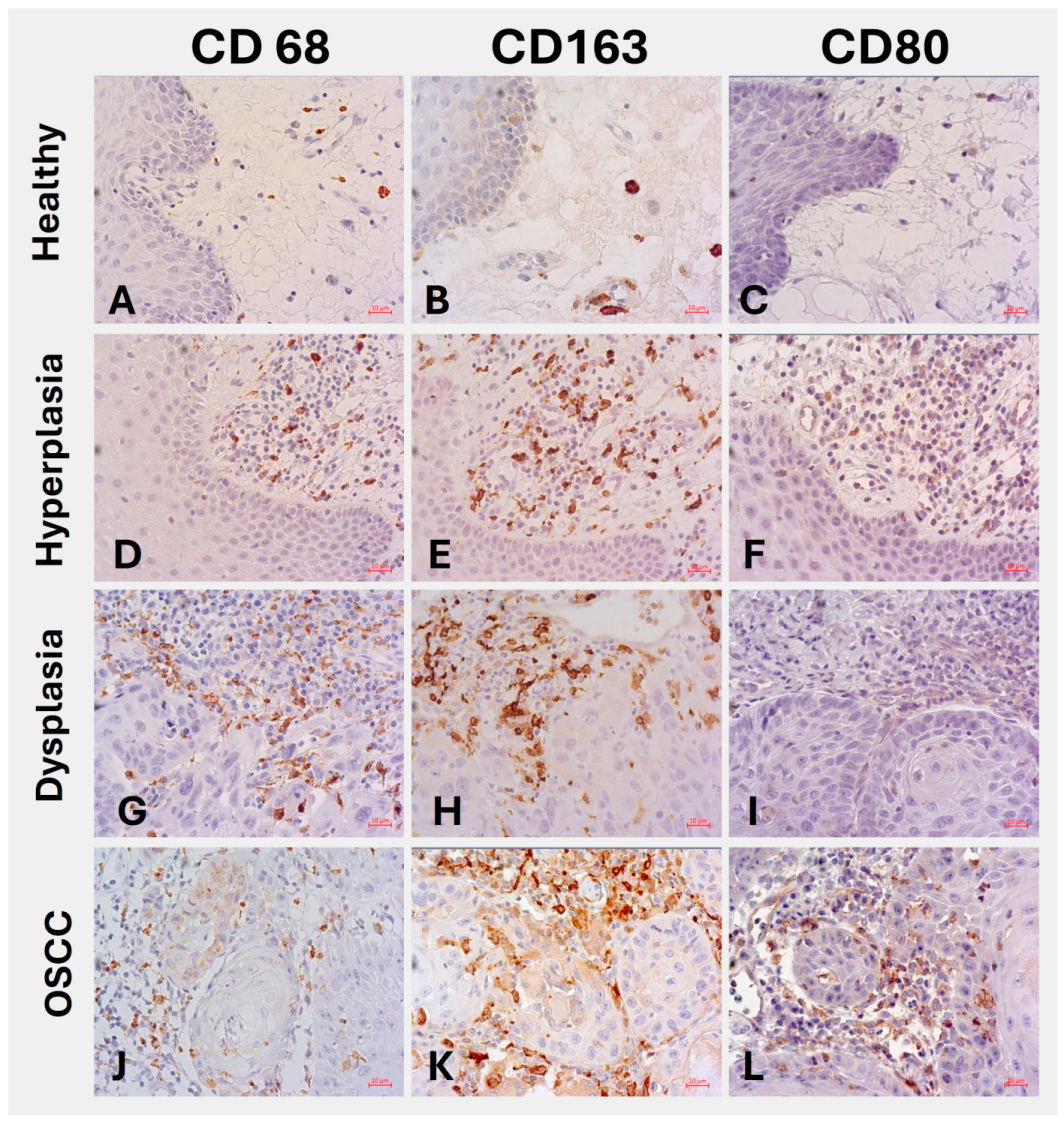
Immunohistochemical expression of macrophage markers in oral tissues (400×). Representative micrographs from the healthy (**A**–**C**), hyperplasia (**D**–**F**), dysplasia (**G**–**I**), and oral squamous cell carcinoma (OSCC) (**J**–**L**) study groups.

**Figure 2:**
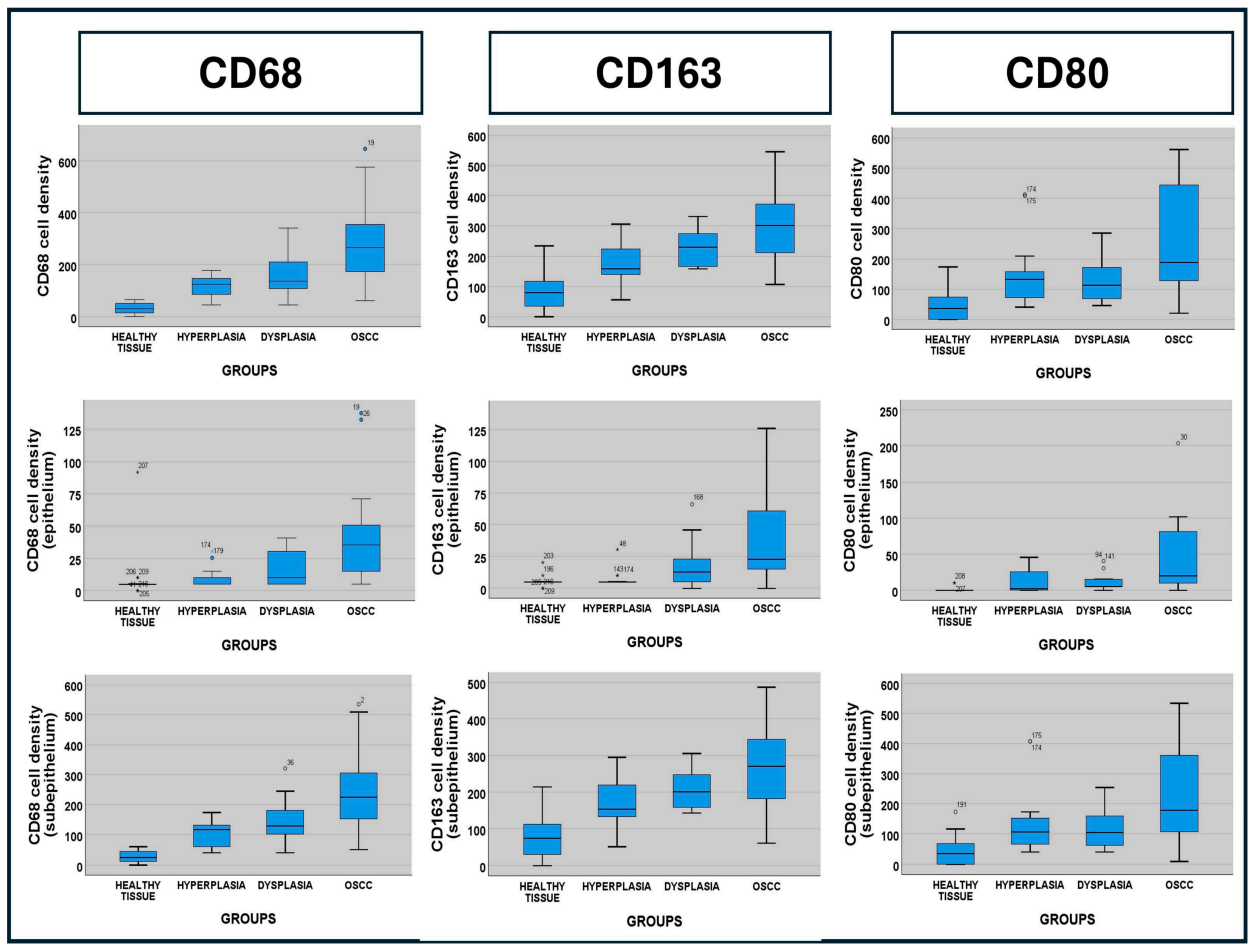
Density (cells/mm^2^) of CD68+, CD163+, and CD80+ macrophages across the four diagnostic groups (healthy tissue, hyperplasia, dysplasia, and OSCC). Columns correspond to the markers CD68, CD163, and CD80; rows correspond to the total, epithelial, and subepithelial compartments. Boxes represent the interquartile range, the horizontal line the median, and whiskers the range; circles and asterisks denote outliers.

A progressive increase in the density of CD68+, CD163+, and CD80+ macrophages was observed across the sequence comprising normal mucosa, OL with hyperplasia, OL with dysplasia, and OSCC in all evaluated compartments (epithelial, subepithelial and both), with significant overall differences for all markers (*p* < 0.001; Table 2; Figure 1 and Figure 2).

For all markers, the density in the subepithelial compartment was substantially higher than that observed in the epithelial compartment in adjusted pairwise comparisons (Table 3).

Regarding CD68 (a pan-macrophage marker), the total density increased from 30.57 cells/mm^2^ in normal mucosa to 264.90 cells/mm^2^ in OSCC. The differences were significant between normal mucosa and dysplasia (*p* = 0.001), normal mucosa and OSCC (*p* < 0.001), and hyperplasia and OSCC (*p* = 0.002), but were not significant between normal mucosa and hyperplasia (*p* = 0.056) or between dysplasia and OSCC (*p* = 0.066). In the epithelial compartment, the density remained low up to the dysplasia (10.19 cells/mm^2^) and increased markedly in OSCC (35.66 cells/mm^2^); this was the only transition for which the difference between dysplasia and OSCC was significant (*p* = 0.019). The pattern in the subepithelial compartment mirrored that observed for total density.

CD163 (an M2 marker) had the highest basal density among the three markers in normal mucosa (78.96 cells/mm^2^, total) and increased progressively to 300.56 cells/mm^2^ in OSCC. In terms of total density, the differences were significant between normal mucosa and dysplasia (*p* = 0.002), normal mucosa and OSCC (*p* < 0.001), and hyperplasia and OSCC (*p* = 0.024). In the subepithelial compartment, the difference between normal mucosa and dysplasia was significant (*p* = 0.002), although the comparison between hyperplasia and OSCC did not reach statistical significance (*p* = 0.192); in the epithelial compartment, only the comparisons involving OSCC were significant.

CD80 (an M1 marker) increased overall throughout the sequence (from 35.66 to 188.49 cells/mm^2^, total), although the density in dysplasia (112.08 cells/mm^2^) was slightly lower than that in hyperplasia (132.45 cells/mm^2^). In terms of total density, significant differences were observed between the normal mucosa and hyperplasia (*p* = 0.032) and between the normal mucosa and OSCC (*p* < 0.001), but not between the normal mucosa and dysplasia (*p* = 0.060). In the epithelial compartment, significant differences were observed between normal mucosa and dysplasia (*p* = 0.046), between the normal mucosa and OSCC (*p* < 0.001), and between the hyperplasia and OSCC (*p* = 0.024). Detailed comparisons for all markers and compartments are presented in Table 3.

### 2.3. Macrophage Polarization Ratios

The CD163/CD68 ratio (an indicator of M2 polarization) decreased throughout the sequence in both the total and subepithelial compartments, whereas the ratios associated with the M1 phenotype (CD80/CD68 and CD80/CD163) increased only in the epithelial compartment; CD80 ratios in the total and subepithelial compartments did not change significantly (Table 4).

The CD163/CD68 ratio decreased from 2.98 in normal mucosa to 1.18 in OSCC in the total compartment (*p* = 0.001) and from 3.40 to 1.17 in the subepithelial compartment (*p* < 0.001). In pairwise comparisons (Table 5), this reduction was significant between normal mucosa and OSCC (total, *p* = 0.004; subepithelial, *p* < 0.001) and in the subepithelial compartment, also between hyperplasia and OSCC (*p* = 0.022). In the epithelial compartment, this ratio remained at 1.00 in all groups, with no significant differences (*p* = 0.891).

The CD80/CD68 ratio did not vary significantly in the total (*p* = 0.473) and subepithelial (*p* = 0.283) compartments, but it increased significantly in the epithelial compartment (from 0.00 in normal mucosa to 0.72 in OSCC; *p* = 0.002), with significant differences between normal mucosa and dysplasia (*p* = 0.011) and between normal mucosa and OSCC (*p* = 0.001). Similarly, the CD80/CD163 ratio varied significantly only in the epithelial compartment (from 0.00 to 0.84; *p* = 0.009), with a significant difference between normal mucosa and OSCC (*p* = 0.004). No significant variation was observed in either the total (*p* = 0.119) or the subepithelial (*p* = 0.224) compartments. Detailed pairwise comparisons are shown in Table 5.

### 2.4. Macrophage Density and Dysplasia Grade in Oral Leukoplakias

In cases of oral leukoplakia (OL), macrophage density and polarization rates were analyzed according to the degree of epithelial dysplasia, using both the three-level WHO classification (mild, moderate, and severe) and the binary histopathological risk classification (low vs. high risk) (Table S1). Most markers, compartments, and rates showed no significant association with the degree of dysplasia. The only exception was the epithelial density of CD68+ macrophages, which increased significantly with the degree of dysplasia in both classifications (three-level WHO classification, *p* = 0.013; binary low vs. high-risk classification, *p* = 0.035). In the three-level classification, this increase was driven by the group with severe dysplasia (median of 30.57 cells/mm^2^) compared with the groups with mild (5.09 cells/mm^2^) and moderate (5.09 cells/mm^2^) dysplasia; in paired comparisons (Table S2), differences were significant between mild and severe cases (*p* = 0.030) and between moderate and severe cases (*p* = 0.045), but not between mild and moderate cases (*p* = 1.000). No significant differences were observed for CD163, CD80, or any of the polarization ratios in any compartment.

### 2.5. Macrophage Density and Grading Systems in OSCC

In the OSCC group, macrophage density and polarization rates were compared across histological grades, (Broders’ classification, infiltration/growth pattern, and the Brandwein–Gensler histological risk model) and the depth of invasion (DOI). No significant differences were found for any marker, compartment, or rate between histological grades (G1–G3; Table S3) or according to the IFG or DOI scores (Table S4). In contrast, the HRM score showed a significant association with macrophage density (Table S4): CD68+ and CD163+ macrophages were significantly more abundant in low-risk tumors than in high-risk tumors, both in the total compartment (CD68, 264.90 vs. 168.11 cells/mm^2^, *p* = 0.012; CD163, 310.75 vs. 198.67 cells/mm^2^, *p* = 0.009) and in the subepithelial compartment (CD68, 234.33 vs. 152.83 cells/mm^2^, *p* = 0.007; CD163, 290.37 vs. 124.30 cells/mm^2^, *p* = 0.007). No significant differences were observed for CD80 or for any of the polarization rates.

### 2.6. Malignant Transformation-Free Survival Analysis

**Figure 3:**
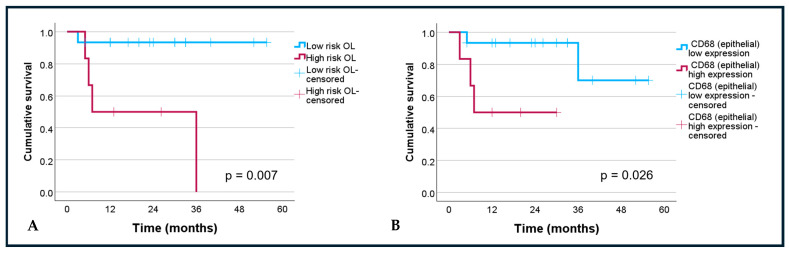
Kaplan–Meier analysis of malignant transformation-free survival (MTFS). (**A**) MTFS according to the binary histological risk classification, showing significantly longer survival in the low-risk group than in the high-risk group. (**B**) MTFS according to epithelial CD68 expression, showing significantly longer survival in lesions with low CD68 expression (<10.2 cells/mm^2^) than in those with high expression (≥10.2 cells/mm^2^).

Among the OL with follow-up information (*n* = 21), five (23.8%) underwent malignant transformation during follow-up (median of 23 months; range 3–56 months). Kaplan–Meier analysis demonstrated a significant difference in malignant transformation-free survival (MTFS) according to the binary histological risk classification (log-rank *p* = 0.007; Figure 3; Supplemental Table S5). Epithelial CD68 expression was also significantly associated with MTFS (log-rank *p* = 0.026; Figure 3; Supplemental Table S5). No statistically significant differences in MTFS were observed according to the other variables (Supplemental Table S5).

In the Cox proportional hazards model, high-risk classification was associated with a higher hazard of malignant transformation compared with low-risk classification (HR = 9.40; 95% CI, 1.04–85.35; *p* = 0.046) (Supplemental Table S6). Epithelial CD68 expression at or above the median was associated with an estimated HR of 6.82, although this association did not reach statistical significance after adjustment for OL histopathological risk classification (95% CI, 0.70–66.49; *p* = 0.098).

## 3. Discussion

### 3.1. Frequency of Expression of the Three Markers in Normal Tissue, Premalignant Lesions, and OSCC

The polarization of tumor-associated macrophages between the M1 phenotype, classically associated with antitumor activity, and the M2 phenotype, generally linked to tumor promotion, has been proposed to contribute to the progression of oral potentially malignant disorders [20]. In this context, the analysis of CD68, CD80, and CD163 expression in the samples studied revealed a progressive increase in the density of all three markers along the normal mucosa, OL (with hyperplasia, with dysplasia), carcinoma sequence, with the highest values observed in OSCC. These findings can be interpreted in light of previously reported data, which describe distinct patterns of macrophage infiltration depending on the studied macrophage phenotype.

Regarding total macrophages identified by CD68, CD68+ density in the present study increased progressively, from 30.57 cells/mm^2^ in normal mucosa to 264.90 cells/mm^2^ in OSCC (in the total compartment), with the difference relative to normal mucosa becoming statistically significant at the dysplasia stage (*p* = 0.001). Previous studies have described a residual presence of CD68-positive macrophages in normal oral mucosa, with infiltration increasing during the progression from normal mucosa to carcinoma [21,22,23]. However, clinicopathological correlations for this marker remain inconsistent. Our findings are consistent with this lack of a clear pattern: CD68+ density was not associated with the histological grade of OSCC, the infiltration/growth pattern (IFG), or the depth of invasion (DOI). The only significant associations involving CD68 were a higher epithelial density with increasing dysplasia grade in oral leukoplakias and, among carcinomas, a higher density in tumors classified as low risk according to the histologic risk model (HRM). Some studies report no association with the degree of dysplasia or malignancy [7,21], while others link CD68 expression to lymph node status [22]. This variability may reflect the heterogeneous composition of the CD68-positive population, which includes M1, M2, and undifferentiated monocyte/macrophage subpopulations [21]. A systematic review also pointed out that CD68 is not a marker specific to macrophages, since it can also mark fibroblasts and tumor cells, which may partly explain the inconsistent clinicopathological correlations reported for this marker [24]. Moreover, an alternative hypothesis is that macrophages play a more prominent role during the early stages of carcinogenesis but contribute less to OSCC progression, particularly in relation to histopathological indicators such as tumor grade. This may suggest that other cellular components of the tumor microenvironment exert a greater influence on disease progression than macrophages. Future studies should therefore investigate the mechanistic roles and dynamic interactions of these cell populations, including tumor-infiltrating lymphocytes (TILs), cancer-associated fibroblasts (CAFs), and other immune and stromal cells.

For M1 macrophages identified by CD80 expression, CD80+ density increased overall along the sequence (from 35.66 to 188.49 cells/mm^2^, total), reaching its highest value in OSCC, although without significant variation between hyperplasia and dysplasia. As reported for CD68, CD80 expression is limited in normal mucosa and, although detectable in OPMDs, does not appear to show significant variation across different grades of dysplasia [7,21]. Importantly, CD80 expression differed significantly between normal oral tissues and hyperplastic oral leukoplakia. Further studies are needed to elucidate the biological significance of this finding and determine whether CD80 may serve as a useful biomarker for these lesions. In carcinoma, CD80-positive macrophages are rare and tend to decrease in higher-grade tumors, although this trend does not reach statistical significance [21]. In contrast to these reports, in our cohort, CD80+ density increased along the sequence and was highest in OSCC, rather than decreasing in carcinoma.

With respect to M2 macrophages identified by CD163, CD163+ density showed the most pronounced progressive increase among the three markers, from 78.96 cells/mm^2^ in normal mucosa to 300.56 cells/mm^2^ in OSCC (total), with the difference relative to normal mucosa already significant at the dysplasia stage (*p* = 0.002). Among the markers examined, CD163 most consistently reflected lesion progression, with expression increasing from minimal levels in normal mucosa to maximal densities in carcinoma [21,22,23]. This upregulation was apparent even at the premalignant stage, as mild to moderate dysplasia showed a significant increase compared with lesions without dysplasia [7]. In carcinoma, CD163-positive macrophage density correlates with histological grade of malignancy [21], although this association has not been consistently observed across all studies [22]. Consistent with these reports, in the present study, CD163 was the marker whose absolute density best reflected lesional progression, reaching its maximal density in OSCC.

### 3.2. Macrophage Polarization in Tumor Progression

The polarization of undifferentiated macrophages (M0) into M1 or M2 phenotypes is determined by the tumor microenvironment and has been suggested an important immunological parameter in tumor progression. Since the isolated quantification of a single marker does not reflect the state of polarization, the ratio of CD163+ cells to CD68+ cells has been used as an indicator of the degree of M2 polarization [25]. Using the same CD163/CD68 ratio, it was found that M2 polarization increases as the lesion progresses, reaching its maximum value in carcinoma [23]. Similarly, in models of progression from normal oral mucosa to carcinoma, the proportion of M2 macrophages increases significantly, alongside changes in T-lymphocyte infiltration [26]. We could not find, however, a significant impact of M1/M2 ratio in our sample, although we report an increase in all macrophage biomarkers, in particular the CD163, especially in OSCC cases. The progressive predominance of the M2 phenotype throughout the sequence is documented [23]. Our findings may reflect the considerable functional plasticity of macrophages within the tumor microenvironment, where activation states exist along a dynamic continuum rather than as two clearly distinct M1 and M2 phenotypes. Polarization toward the M2 phenotype does not occur in isolation; rather, it is modulated by changes in the lymphocyte compartment, with which macrophages establish bidirectional, cytokine-mediated communication. Interleukins from T helper cells determine the direction of polarization as follows: IFN-γ from Th1 cells favors the antitumor M1 phenotype, while Th2 cytokines, such as IL-4, IL-10, and IL-13, promote M2 polarization [27]. This balance shifts as the lesion progresses: in potentially malignant lesions, CD8^+^ T lymphocytes are abundant and the CD8^+^/CD4^+^ ratio increases following dysplasia, but as the lesion progresses, a tolerance profile sets in, with a predominance of CD25^+^FoxP3^+^CD4^+^ regulatory T cells, which, by secreting IL-10 and TGF-β1, promote pro-tumor immunity [9]. This progression is reflected in the correlation between the two populations: CD163^+^ M2 macrophages increase in parallel with regulatory T cells and IL-10 throughout the sequence [28], correlating with CD4^+^ lymphocytes in a Th1 microenvironment during the premalignant phase [7], a relationship that weakens in carcinoma [21], where the proportion of M2 macrophages is nearly double that of M1 macrophages [16].

This macrophage–lymphocyte crosstalk also influences immune checkpoint regulation. M2-polarized TAMs express PD-L1, which inhibits T-cell activation and contributes to local immunosuppression. In OSCC, the density of CD163^+^ macrophages correlates with tumoral PD-L1 expression, linking M2 polarization to disease progression and highlighting the functional impact of the immunosuppressive microenvironment [29,30].

### 3.3. Association with Clinicopathological Variables

In the present study, macrophage infiltration showed few associations with the clinicopathological characteristics of the lesions. In the oral squamous cell carcinoma (OSCC) group, neither the densities of CD68+, CD163+, and CD80+ macrophages nor the polarization ratios differed significantly among histological grades (G1–G3), the infiltration/growth pattern (IFG), or the depth of invasion (DOI). This lack of association is consistent with the inconsistent prognostic behavior reported for the pan-macrophage marker CD68 in OSCC: a recent meta-analysis concluded that CD68+ tumor-associated macrophages (TAMs) were not associated with overall survival, whereas only the CD163+ (M2) subset retained prognostic value [31]. Other authors have reported the opposite: a high density of CD68+ macrophages was associated with worse survival [15]. These discrepancies suggest that the total macrophage population, as well as morphology-based classification systems themselves, may not adequately reflect the functional state of the tumor’s immune microenvironment.

In oral leukoplakia, the only significant finding was an increase in the epithelial density of CD68+ macrophages with the degree of dysplasia, reaching its highest value in severe dysplasia, with no change in the CD163+ or CD80+ populations. This early and compartment-specific increase is consistent with previous reports showing that macrophage infiltration progressively increases from normal mucosa, through dysplasia, to invasive carcinoma [22], and suggests that macrophage recruitment to the epithelium accompanies the early stages of oral carcinogenesis, before a polarization profile is established.

Among the classification systems applied to oral squamous cell carcinoma (OSCC), only the histologic risk model (HRM) showed a significant association with macrophage density: CD68+ and CD163+ macrophages were more abundant in low-risk tumors than in high-risk tumors, both in the total compartment and in the subepithelial compartment. This result is, at first glance, counterintuitive, given that the prevailing literature associates a high density of tumor-associated macrophages (TAMs), particularly the CD163+ population, with reduced survival in OSCC [22,31]. As stated before, macrophages could play a more prominent role during the early stages of carcinogenesis but contribute less to OSCC progression. One possible explanation may lie in the HRM classification itself, which incorporates the host’s lymphocytic response as a scoring parameter [32]. Tumors that trigger a more intense host immune response may receive a lower risk score precisely because of this response. According to this interpretation, the higher macrophage density in low-risk tumors would reflect a more active immune microenvironment, rather than a pro-tumor one. This hypothesis remains speculative and, given the limited number of cases per HRM category, requires confirmation in larger cohorts. In another study, which evaluated 125 OSCC samples, no associations were found with most of the clinicopathological variables or with survival. That same study evaluated each marker separately and did not calculate polarization ratios, unlike the present study, in which the CD163/CD68, CD80/CD68, and CD80/CD163 ratios were used as indicators of macrophage polarization [30].

### 3.4. Macrophages as Prognostic Factors and Therapeutic Targets in OSCC

Tumor-associated macrophages have been investigated as prognostic biomarkers in OSCC, with a consistent pattern: the pan-macrophage marker CD68 has no prognostic value, whereas CD163^+^ M2 macrophages are associated with poorer survival [31,33]. CD163^+^ M2 macrophages have, in fact, been identified as one of the most promising predictors of survival in oral cancer [34], reinforcing that it is the characterization of the M2 phenotype and not the isolated quantification of CD68 that has greater prognostic value in OSCC [33,34].

This significance makes M2 macrophages an attractive therapeutic target through the following three main approaches: blocking their recruitment to the tumor (notably by inhibiting the CCL2/CCR2 and CSF-1/CSF-1R axes), depletion of existing populations, and their repolarization toward the antitumor M1 phenotype [35]. In head and neck cancer, these strategies have been proposed primarily in combination with PD-1/PD-L1 checkpoint inhibition [36]. In this context, the IL-17 axis stands out for its link to M2 polarization: IL-17 promotes the infiltration of the carcinoma by M2 macrophages via STAT3 [37], and its inhibition has delayed tumor development and prolonged survival in animal models, supporting the exploration of IL-17 blockade as a therapeutic strategy in OSCC [37].

### 3.5. Malignant Transformation-Free Survival Analysis of OL Cases

Both the binary histological risk classification and epithelial CD68-positive macrophage infiltration were significantly associated with malignant transformation-free survival (MTFS). High-risk lesions had shorter MTFS, consistent with the reports of Kujan et al. [38] who proposed the binary system for predicting malignant transformation in comparison to the WHO dysplasia classification 2005 [39].

The association between high epithelial CD68 and shorter MTFS in univariate analysis agrees with Weber et al. [23] who observed increased epithelial CD68-positive macrophage infiltration in 82.0% of transforming leukoplakia’s compared with 54.7% of non-transforming lesions. No other macrophages markers were related with MTFS. Yagyuu et al. [40] reported that increased subepithelial CD163-positive macrophages were associated with high-grade dysplasia and malignant-free survival. These findings support again the involvement of macrophages in the early stages of oral carcinogenesis. However, larger prospective studies are required to confirm its independent prognostic value.

### 3.6. Limitations and Future Perspectives

This study has some limitations. The sample size was relatively small, particularly in the carcinoma and OPMD groups, which may restrict the statistical power and generalizability of the results. Furthermore, the retrospective nature of the study limited the availability of clinicopathological and follow-up data, which were incomplete in some cases, thereby preventing a more in-depth analysis of the association between the markers and clinical behavior or prognosis. The limited number of cases with adequate follow-up and documented malignant transformation events should also be acknowledged. Added to this are the technical variability and subjectivity inherent in immunohistochemical assessment, which are not always fully controllable. Nevertheless, controls were consistently included on additional slides and within each TMA as internal controls for all markers. The biological and prognostic significance of macrophages may also vary according to their microlocalization, by influence of other cells of immune microenvironment and differences in the anatomical areas selected for quantification may partly explain potential discrepancies among previous studies [41,42]. There is also a conceptual limitation: the M1 and M2 phenotypes represent the extremes of a continuous spectrum rather than discrete categories, and CD163, although widely used as a marker of M2 macrophages, is not exclusive to this phenotype, which may introduce some imprecision in quantification. In addition, the present study focused on TAMs and did not characterize the wider immune microenvironment.

The findings of this study suggest multiple directions for future research. Future studies using broader immune panels, including multiplex immunohistochemistry or spatial immune profiling, may complement these findings and further clarify the interactions between macrophages and other immune-cell populations. Validation in larger, prospective cohorts, with correlation to clinical follow-up data, is necessary to clarify the prognostic significance of M2 macrophages and the polarization ratio in OSCC. The focus lies in the therapeutic aspect. Given the role of M2 macrophages in tumor progression, it is important to investigate strategies that target them by inhibiting their recruitment or repolarizing them toward the M1 phenotype [35], combined with PD-1/PD-L1 checkpoint inhibition [36]. The IL-17 axis is also emerging as a target because of its association with M2 polarization and the promising results of its blockade in animal models [37].

## 4. Materials and Methods

### 4.1. Design of the Study and Ethical Considerations

This was a retrospective observational cohort study performed in the Research unit of UNIPRO at IUCS university direct to test the research hypothesis of an existing association of an immunophenotype macrophage expression in oral carcinogenesis characteristic lesions. This study was conducted in accordance with the ethical principles outlined in the Declaration of Helsinki and was approved by the Ethics Committee of the Instituto Universitário de Ciências da Saúde, Portugal (approval no. 41CE-UICS2025; date of approval: 18 December 2025). All patient data were handled in accordance with confidentiality standards, and all samples were anonymized, ensuring that no identifying information was disclosed.

### 4.2. Sample Collections and Selections

We include cases of patients with primary diagnosis of oral leukoplasias, oral squamous cell carcinoma (OSCC) and healthy tissues all located in the oral cavity (ICD 10: C00-06) from the archives of the Histopathology Lab of UNIPRO research center at IUCS-CESPU, Portugal, from the period of 2001 to 2026.

Clinical and pathological characteristics data were retrieved. All diagnoses were confirmed based on histopathological examination. Inclusion criteria required histologically confirmed diagnosis, sufficient, and well-preserved formalin-fixed, paraffin-embedded (FFPE) tissue samples for immunohistochemical analysis. Samples with inadequate tissue, poor preservation, or incomplete relevant clinical data were excluded. Patients younger than 18 years old, patients that had oral cancers previously or had received radiotherapy or chemotherapy for head and neck cancers were also excluded. A total of 81 cases were included in the study, comprising 16 normal oral mucosa, 14 oral leukoplakias with either epithelial hyperplasia or hyperkeratosis/or parakeratosis without dysplasia and 16 OL with epithelial dysplasia, and 35 OSCC samples.

Histopathological evaluation was conducted on new 3 µm sections stained with hematoxylin and eosin (H&E).

Oral leukoplakia (OL) was diagnosed according to the classification criteria established by Warnakulasuriya et al. [10]. Furthermore, epithelial dysplasia was evaluated and graded by two experienced pathologists following the 5th edition of the WHO Classification of Head and Neck Tumors. These lesions were stratified into three severity tiers, mild, moderate, and severe, based on the degree of architectural and cytological changes [43], as well as binary histopathological risk classification according to Warnakulasuriya et al. [39] (low-risk and high-risk OL).

Oral squamous cell carcinoma (OSCC) diagnoses were confirmed following the 5th edition of the World Health Organization (WHO) Classification of Head and Neck Tumors. Histopathological grading was performed using the classic Broders’ system, which classifies tumors as well-differentiated (Grade 1), moderately differentiated (Grade 2), or poorly differentiated (Grade 3). Furthermore, we applied the invasive front grading (IFG) system, which assesses the tumor-host interface by scoring the following four parameters: keratinization, nuclear polymorphism, mitotic activity, and lymphoplasmacytic response. Tumors were also stratified using the Brandwein–Gensler histologic risk model (HRM), which assigns risk levels (low, intermediate, or high) based on the combined assessment of the worst pattern of invasion (WPOI), lymphocytic host response (LHR), and perineural invasion (PNI). Depth of invasion (DOI) was recorded in millimeters (mm), defined as the perpendicular distance from the basement membrane of the adjacent normal mucosa to the deepest point of tumor infiltration. Additionally, we documented the presence of vascular, muscular, and bone invasion, as well as nodal metastasis. In instances of inter-observer diagnostic discrepancy, a consensus was reached. For cases presenting with multiple lesions or samples, the most advanced and representative lesion was selected for histopathological grading and biomarker analysis.

### 4.3. Tissue Processing and TMA Construction

Immunohistochemical analysis was performed using tissue microarray (TMA) sections with three cylindrical tissue cores (2 mm in diameter) from each selected specimen constructed. Areas of representative lesions were identified on hematoxylin-and-eosin-stained slides whole-tissue sections previously selecting the most aggressive or invasive front areas. Sections of lymph nodes and tonsils were used as positive controls for these cases.

### 4.4. Immunohistochemical Staining

#### 4.4.1. Antigen Retrieval

TMA sections were first deparaffinized in xylene and rehydrated through a graded series of decreasing alcohol concentrations, followed by rinsing in distilled water. Antigen retrieval was performed using a heat-induced epitope retrieval method. For CD163, retrieval was carried out in EDTA buffer, while CD68 required citrate buffer. For CD80, antigen retrieval was performed using the IHC-Tek Steamer with the EnVision Flex Mini Kit (high pH) according to the manufacturer’s instructions.

#### 4.4.2. Immunohistochemical Staining Protocol

Immunohistochemical staining was performed in accordance with the respective manufacturers’ instructions using the Envision Flex Mini Kit (Dako/Agilent) detection systems. Following antigen retrieval and the blocking steps, the sections were incubated with the primary antibodies according to the optimized dilution and incubation time for each marker: CD68 (1:100, 1 h), CD163 (1:100, 1 h), and CD80 (1:100, 1 h and 30 min). Detection was performed using the previous detection system and the immunoreaction was visualized with 3,3′-diaminobenzidine (DAB) for 7 to 10 min, at a dilution of 50:1000 for CD68 and CD163 and 1 drop of DAB per 1000 of solvent for CD80. Finally, the sections were stained with hematoxylin, dehydrated, cleared, and mounted. The primary antibodies used are summarized in Table 6.

### 4.5. Evaluation of Immunostaining

Immunohistochemical evaluation was conducted independently by two authors blinded to clinical variables. In the presence of a discordant result between the two authors’ evaluation, a review of the slides was performed under a multi-head microscope to achieve a consensus.

For each marker (CD68, CD163, and CD80), only visible staining compatible with cell staining were classified as positive. Macrophage density was assessed by counting CD68+, CD163+, and CD80+ cells in five representative high-power fields (HPF, 400× magnification) per specimen. To ensure standardized quantification, images were captured using a ZEISS AxioLab A1^®^ microscope (Carl Zeiss Microscopy GmbH, Jena, Germany), with a ZEISS Axiocam 105 color^®^ and ZEISS Zen version 3.13^®^ software equipped with a 40× objective and a 10×/FN20 eyepiece, resulting in a fixed field area of approximately 0.196 mm^2^ per field.

For each marker, cell density was quantified separately in the epithelial and subepithelial tissue compartments, allowing the assessment of macrophage localization across the epithelial–stromal interface.

In addition, the CD163/CD68, CD80/CD68 and CD80/CD163 ratios were calculated for each case based on the respective number of positive cells, as indicators of macrophage polarization. All evaluations were performed under standardized conditions and predefined scoring criteria to minimize assessment bias.

### 4.6. Statistical Analysis

All statistical analyses were performed using IBM SPSS Statistics software version 29.0 (IBM Corp., Armonk, NY, USA). Continuous data regarding macrophage density and the M1/M2 ratio were expressed as median and interquartile range (IQR), as they deviated from a normal distribution according to the Shapiro–Wilk test.

The demographic and clinical characteristics of the study population were compared across the four diagnostic groups (healthy tissue, OL without dysplasia, OL with dysplasia, and OSCC). The Kruskal–Wallis test was used for age and histological grade assessment. Categorical data, such as gender and lesion site, were compared using the Chi-square test or Fisher’s exact test when the expected frequencies were less than five.

To evaluate differences in the expression of CD68, CD163, and CD80 markers across the four study groups, the Kruskal–Wallis test was employed. Significant results were followed by Dunn’s post hoc test with Bonferroni correction for multiple comparisons. Comparisons between independent binary variables (e.g., gender) and macrophage density were performed using the Mann–Whitney U test.

For survival analyses, malignant transformation-free survival (MTFS) was defined as the time from study entry to malignant transformation, with non-transformed lesions censored at the last follow-up. MTFS was estimated using the Kaplan–Meier method and compared using the log-rank test. Variables significantly associated with MTFS in Kaplan–Meier analysis were entered into a multivariable Cox regression model, and results were reported as hazard ratios with 95% confidence intervals and *p*-values.

A *p*-value of <0.05 was considered statistically significant for all analyses.

## 5. Conclusions

In this observational study, the density of CD68+, CD163+, and CD80+ macrophages increased progressively along the sequence from normal mucosa through hyperplasia, and dysplasia to OSCC, in both compartments analyzed, and was consistently higher in the subepithelial compartment. Among the three markers, CD163+ M2 macrophages reached the highest absolute density in OSCC, confirming their accumulation throughout oral carcinogenesis. Significant differences were observed in CD80+ cell density between healthy mucosa and hyperplasia and between dysplasia and OSCC, as well as in epithelial CD68+ macrophage density between moderate and severe dysplasia.

However, the CD163/CD68 ratio, used as an indicator of M2 polarization, did not show an increase trend along the sequence. This could be an indication that the expansion of the M2 population was accompanied by probably other immune cell population, rather than by a growing relative predominance of the M2 phenotype.

Therapeutic modulation of M2 macrophages remains a promising avenue that warrants further investigation. Given the prognostic significance attributed to M2 macrophages in OSCC, validation in larger, prospective cohorts with clinical follow-up would be indicated.

## Figures and Tables

**Table 1:** Distribution of the cases by clinical and pathological variables across the study groups.

Variable	Total (*n* = 81)	Healthy Tissue (*n* = 16)	OL with Hyperplasia (*n* = 14)	OL with Dysplasia (*n* = 16)	OSCC (*n* = 35)	*p*-Value
**Age (median, IQR)**	62.0 (50.0–75.5)	63.5 (55.0–69.8)	71.5 (48.0–79.0)	46.5 (43.3–69.8)	61.0 (54.0–77.0)	0.226
Sex, *n* (%)						0.344
Female	27 (33.3)	5 (31.3)	7 (50.0)	3 (18.8)	12 (34.3)	
Male	54 (66.7)	11 (68.8)	7 (50.0)	13 (81.3)	23 (65.7)	
**Location,** *n* (%)						0.055
Tongue/floor of mouth	19 (23.5)	0 (0.0)	4 (28.6)	3 (18.8)	12 (34.3)	
Others	62 (76.5)	16 (100.0)	10 (71.4)	13 (81.3)	23 (65.7)	
**Dysplasia grade,** *n* (%)						
Mild	4 (4.9)	–	–	4 (25.0)	–	–
Moderate	6 (7.4)	–	–	6 (37.5)	–	–
Severe	6 (7.4)	–	–	6 (37.5)	–	–
**Binary histopathological risk classification,** *n* (%)						
Low risk	18 (60%)	–	14 (100.0)	4 (25.0)	–	–
High risk	12 (40%)	–	–	12 (75.0)	–	–
**Histological grade,** *n* (%)						
Well diff. (G1)	20 (57.1)	–	–	–	20 (57.1)	–
Mod. diff. (G2)	9 (25.7)	–	–	–	9 (25.7)	–
Poorly diff. (G3)	6 (17.1)	–	–	–	6 (17.1)	–
**HRM score (OSCC),** *n* (%)						
Score 1	27 (77.1)	–	–	–	27 (77.1)	
Score 2	8 (22.9)	–	–	–	8 (22.9)	
**IFG score (OSCC),** *n* (%)						
Score 1	18 (51.4)	–	–	–	18 (51.4)	
Score 2	17 (48.6)	–	–	–	17 (48.6)	

IQR, interquartile range; OL, oral leukoplakia; OSCC, oral squamous cell carcinoma; HRM, histologic risk model; IFG, invasive front grading.

**Table 2:** Macrophage density (cells/mm^2^) by group of lesions, expressed as median (IQR).

Marker/Compartment	Healthy (Median/IQR)	OL/Hyperplasia (Median/IQR)	OL/Dysplasia (Median/IQR)	OSCC (Median/IQR)	*p*-Value
**CD68 Total**	30.57 (12.74–50.94)	124.81 (77.69–150.28)	137.54 (105.70–215.23)	264.90 (171.93–357.87)	<0.001
**CD68 Epithelial**	5.09 (5.09–5.09)	5.09 (5.09–11.46)	10.19 (5.09–30.57)	35.66 (14.01–52.22)	<0.001
**CD68 Subepithelial**	25.47 (11.46–45.85)	117.17 (56.04–135.00)	129.91 (99.34–184.67)	224.15 (152.83–305.65)	<0.001
**CD163 Total**	78.96 (35.66–122.27)	157.92 (139.45–234.33)	229.24 (161.74–282.73)	300.56 (208.23–380.80)	<0.001
**CD163 Epithelial**	5.09 (5.09–5.09)	5.09 (5.09–6.37)	12.74 (5.09–24.20)	22.93 (14.01–61.13)	<0.001
**CD163 Subepithelial**	73.87 (30.57–117.17)	152.83 (131.81–222.87)	199.95 (155.38–250.89)	269.99 (175.49–351.37)	<0.001
**CD80 Total**	35.66 (0.00–75.14)	132.45 (64.95–170.66)	112.08 (67.50–177.02)	188.49 (126.09–448.29)	<0.001
**CD80 Epithelial**	0.00 (0.00–0.00)	2.55 (0.00–26.75)	5.09 (5.09–15.28)	20.38 (8.92–81.51)	<0.001
**CD80 Subepithelial**	35.66 (0.00–70.05)	106.98 (61.14–157.92)	104.43 (62.41–164.29)	178.30 (105.71–373.15)	<0.001

IQR, interquartile range. OL, oral leukoplakia; OSCC, oral squamous cell carcinoma; *p*-values from the Kruskal–Wallis test.

**Table 3:** Pairwise comparisons (*p*-values) between study groups for macrophage density (corresponding to Table 2).

Group Comparison	CD68 Tot	CD68 Ep	CD68 Sub	CD163 Tot	CD163 Ep	CD163 Sub	CD80 Tot	CD80 Ep	CD80 Sub
**Healthy vs. Hyperplasia**	0.056	1.000	0.056	0.153	1.000	0.090	0.032	0.487	0.034
**Healthy vs. Dysplasia**	0.001	0.366	0.001	0.002	0.083	0.002	0.060	0.046	0.062
**Healthy vs. OSCC**	<0.001	<0.001	<0.001	<0.001	<0.001	<0.001	<0.001	<0.001	<0.001
**Hyperplasia vs. Dysplasia**	1.000	1.000	1.000	1.000	0.558	1.000	1.000	1.000	1.000
**Hyperplasia vs. OSCC**	0.002	<0.001	0.002	0.024	<0.001	0.192	0.285	0.024	1.000
**Dysplasia vs. OSCC**	0.066	0.019	0.086	0.872	0.169	1.000	0.089	0.272	0.459

Asymptotic significances (2-sided tests) are displayed. Significance values were adjusted by the Bonferroni correction for multiple tests. Ep, epithelial; Sub, subepithelial; Tot, total.

**Table 4:** Macrophage immunophenotype ratios by group of lesions, expressed as median (IQR).

Ratio	Healthy (Median/IQR)	OL/Hyperplasia (Median/IQR)	OL/Dysplasia (Median/IQR)	OSCC (Median/IQR)	*p*-Value
**CD163/CD68 Total**	2.98 (1.34–6.10)	1.61 (1.27–1.88)	1.73 (1.05–2.06)	1.18 (0.84–1.53)	0.001
**CD163/CD68 Epithelial**	1.00 (1.00–1.00)	1.00 (0.46–1.00)	1.00 (0.50–3.00)	1.00 (0.51–2.00)	0.891
**CD163/CD68 Subepithelial**	3.40 (1.38–6.79)	1.63 (1.29–2.15)	1.68 (1.14–1.90)	1.17 (0.77–1.50)	<0.001
**CD80/CD68 Total**	1.29 (0.16–3.07)	1.12 (0.98–1.53)	1.05 (0.47–1.35)	0.84 (0.48–1.58)	0.473
**CD80/CD68 Epithelial**	0.00 (0.00–0.00)	0.50 (0.00–4.63)	1.00 (0.17–1.00)	0.72 (0.15–2.06)	0.002
**CD80/CD68 Subepithelial**	1.42 (0.18–3.98)	1.14 (0.87–1.84)	1.05 (0.46–1.35)	0.82 (0.50–1.38)	0.283
**CD80/CD163 Total**	0.42 (0.04–0.94)	0.74 (0.60–0.92)	0.47 (0.29–0.71)	0.76 (0.43–1.32)	0.119
**CD80/CD163 Epithelial**	0.00 (0.00–0.00)	0.50 (0.00–2.13)	0.75 (0.08–1.00)	0.84 (0.25–1.59)	0.009
**CD80/CD163 Subepithelial**	0.44 (0.04–0.97)	0.70 (0.56–0.84)	0.62 (0.31–0.70)	0.72 (0.41–1.40)	0.224

IQR, interquartile range. OL, oral leukoplakia; OSCC, oral squamous cell carcinoma *p*-values from the Kruskal–Wallis test.

**Table 5:** Pairwise comparisons between study groups for macrophage polarization ratios (corresponding to Table 4).

Group Comparison	CD163/ CD68 Tot	CD80/ CD68 Tot	CD80/ CD163 Tot	CD163/ CD68 EP	CD80/ CD68 EP	CD80/ CD163 EP	CD163/ CD68 Sub	CD80/ CD68 Sub	CD80/ CD163 Sub
**Healthy vs. Hyperplasia**	1.000	1.000	1.000	1.000	0.093	0.339	1.000	1.000	1.000
**Healthy vs. Dysplasia**	1.000	1.000	1.000	1.000	0.011	0.161	0.764	1.000	1.000
**Healthy vs. OSCC**	0.004	1.000	0.454	1.000	0.001	0.004	< 0.001	0.635	0.690
**Hyperplasia vs. Dysplasia**	1.000	1.000	0.941	1.000	1.000	1.000	1.000	1.000	1.000
**Hyperplasia vs. OSCC**	0.084	0.841	1.000	1.000	1.000	1.000	0.022	0.858	1.000
**Dysplasia vs. OSCC**	0.082	1.000	0.293	1.000	1.000	1.000	0.068	1.000	0.509

Asymptotic significances (2-sided tests) are displayed. Significance values were adjusted by the Bonferroni correction for multiple tests. Ep, epithelial; Sub, subepithelial; Tot, total.

**Table 6:** Primary antibodies used for immunohistochemical analysis, including antigen, cellular marker, antibody type, dilution and supplier information.

Antigen	Marker	Antibody	Dilution	Vendor
CD68	Pan-macrophage	Mouse anti-human CD68 mAb (clone [LM1])	1:100	Dako^®^, Glostrup, Denmark
CD163	M2 macrophage	Mouse anti-human CD163 mAb (clone 10D6)	1:100	Novocastra^®^, Nussloch, Germany
CD80	M1 macrophage	Rabbit polyclonal anti-CD80 antibody (HPA050092)	1:100	Merck, Darmstadt, Germany

## Data Availability

Research data are available on the request to the corresponding author.

## Supplementary Materials

The following supporting information can be downloaded at https://www.mdpi.com/article/10.3390/ijms27156889/s1.

## Abbreviations

The following abbreviations are used in this manuscript:

CD68	Cluster of differentiation 68 (pan-macrophage marker)
CD80	Cluster of differentiation 80 (M1 macrophage marker)
CD163	Cluster of differentiation 163 (M2 macrophage marker)
DAB	3,3′-Diaminobenzidine
DOI	Depth of invasion
FFPE	Formalin-fixed, paraffin-embedded
HPF	High-power field
HRM	Histologic risk model (Brandwein–Gensler classification)
IFG	Infiltration/growth pattern (Bryne’s classification)
IHC	Immunohistochemistry
IQR	Interquartile range
LHR	Lymphocytic host response
M0	Undifferentiated (non-polarized) macrophage
M1	Classically activated macrophage
M2	Alternatively activated macrophage
OL	Oral leukoplakia
OPMD	Oral potentially malignant disorder
OSCC	Oral squamous cell carcinoma
PD-L1	Programmed death-ligand 1
PNI	Perineural invasion
STAT1	Signal transducer and activator of transcription 1
TAM	Tumor-associated macrophage
Th1	T helper type 1
Th2	T helper type 2
TMA	Tissue microarray
TME	Tumor microenvironment
WHO	World Health Organization
WPOI	Worst pattern of invasion
