# Supplementary material for: Immunophenotypic Characterization of Tumor-Associated Macrophages in Oral Carcinogenesis: An Observational Study
Source: Int J Mol Sci. 2026 Aug 1;27(15):6889. doi: 10.3390/ijms27156889 (PMC13467404; doi:10.3390/ijms27156889)
Supplement: Supplementary file 1 [file ijms-27-06889-s001.zip › ijms-4440049-supplementary.pdf]

Supplemental Table S1. Macrophage immunophenotype density (cell/mm2) and M ratios by dysplasia grade for OL.

	**Three grade dysplasia classification**				**Binary histopathological risk classification**		
**Marker / Compartment**	**Mild (Median / IQR)**	**Moderate (Median / IQR)**	**Severe (Median / IQR)**	**p-value**	**Low (Median / IQR)**	**High (Median / IQR)**	**p-value**
**CD68 Total**	127.36 (59.86–225.42)	127.36 (86.60–154.10)	193.58 (127.36–280.18)	0.189	124.81 (77.69–154.10)	137.54 (117.17–203.77)	0.158
**CD68 Epithelial**	5.09 (5.09–8.92)	5.09 (5.09–15.29)	30.57 (15.29–35.66)	**0.013***	5.09 (5.09–10.19)	10.19 (5.09–30.57)	**0.035***
**CD68 Subepithelial**	119.72 (54.76–219.05)	122.27 (80.24–142.64)	152.83 (106.98–254.71)	0.308	117.17 (56.04–142.64)	127.36 (106.98–173.20)	0.200
**CD163 Total**	201.22 (161.74–305.65)	168.11 (157.92–223.51)	239.43 (231.79–295.47)	0.057	165.56 (145.82–238.16)	229.24 (157.92–259.80)	0.113
**CD163 Epithelial**	17.83 (6.37–56.03)	10.19 (5.09–16.56)	15.28 (7.64–38.21)	0.651	5.09 (5.09–10.19)	15.28 (5.09–20.38)	0.346
**CD163 Subepithelial**	165.56 (151.55–271.27)	163.02 (150.28–206.96)	229.24 (208.87–270.00)	0.060	157.92 (140.09–222.87)	203.77 (152.83–239.43)	0.124
**CD80 Total**	89.15 (52.22–114.62)	129.90 (86.61–205.04)	66.23 (53.49–213.96)	0.376	112.08 (64.95–142.64)	122.26 (66.23–183.39)	0.518
**CD80 Epithelial**	5.09 (1.27–5.09)	15.28 (3.82–19.10)	5.09 (2.55–12.74)	0.259	5.09 (0.00–10.19)	10.19 (5.09–15.28)	0.134
**CD80 Subepithelial**	84.06 (50.95–109.53)	106.98 (85.33–189.76)	61.14 (48.40–203.78)	0.539	106.98 (61.14–137.55)	106.97 (61.14–168.11)	0.723
**CD163/CD68 Total**	1.79 (1.11–3.40)	1.78 (1.09–1.93)	1.50 (1.05–2.10)	0.762	1.61 (1.27–1.93)	1.73 (1.05–2.00)	0.755
**CD163/CD68 Epithelial**	3.00 (1.00–11.01)	1.25 (0.79–3.25)	0.50 (0.26–2.25)	0.162	1.00 (0.87–1.25)	1.00 (0.40–3.00)	0.692
**CD163/CD68 Subepithelial**	1.47 (1.09–3.52)	1.74 (1.12–1.93)	1.87 (1.13–2.14)	0.976	1.63 (1.25–2.15)	1.81 (1.14–1.90)	0.755
**CD80/CD68 Total**	0.76 (0.34–1.43)	1.20 (0.64–1.61)	0.48 (0.26–1.29)	0.365	1.08 (0.81–1.32)	1.06 (0.48–1.35)	0.851
**CD80/CD68 Epithelial**	1.00 (0.25–1.00)	2.00 (0.75–3.00)	0.40 (0.08–0.50)	0.173	1.00 (0.00–1.87)	0.50 (0.17–3.00)	0.692
**CD80/CD68 Subepithelial**	0.76 (0.36–1.48)	1.23 (0.59–1.53)	0.57 (0.28–1.39)	0.550	1.13 (0.69–1.66)	1.20 (0.52–1.35)	1.000
**CD80/CD163 Total**	0.38 (0.31–0.46)	0.70 (0.55–0.97)	0.22 (0.20–0.93)	0.155	0.69 (0.44–0.84)	0.67 (0.22–0.87)	0.755
**CD80/CD163 Epithelial**	0.14 (0.02–0.80)	1.00 (0.56–3.75)	0.17 (0.06–1.00)	0.277	0.14 (0.00–1.04)	1.00 (0.11–1.00)	0.465
**CD80/CD163 Subepithelial**	0.38 (0.32–0.57)	0.67 (0.57–0.85)	0.24 (0.19–0.97)	0.299	0.64 (0.42–0.81)	0.64 (0.24–0.75)	0.851

Statistical significance for the WHO classification (3 grades) was determined using the Kruskal-Wallis test, while the Mann-Whitney U test was employed for the binary risk classification (Low vs. High risk).

Supplemental Table S2. Distribution of p-values within each category of 3 grade dysplasia classification of Table S1.

**Comparison**	**CD68 Total**	**CD68 Epithelial**	**CD68 Subepithelial**	**CD163 Total**	**CD163 Epithelial**	**CD163 Subepithelial**	**CD80 Total**	**CD80 Epithelial**	**CD80 Subepithelial**
**Mild vs. Moderate**	1.000	1.000	1.000	1.000	1.000	1.000	0.486	0.317	0.814
**Moderate vs. Severe**	0.235	**0.045**	0.436	0.051	1.000	0.068	1.000	1.000	1.000
**Mild vs. Severe**	0.665	**0.030**	0.833	0.596	1.000	0.318	1.000	0.695	1.000

Supplemental Table S3. Macrophage immunophenotype density (cell/mm2) and M ratios by histological grade for OSCC.

Marker / Compartment	G1 (Median)	G2 (Median)	G3 (Median)	p-value
CD68 Total	262.35 (164.29 - 352.78)	305.65 (221.60 - 453.39)	224.14 (154.36 - 326.03)	0.382
CD68 Epithelial	35.66 (11.46 - 44.58)	40.75 (28.02 - 61.13)	20.38 (7.64 - 43.30)	0.172
CD68 Subepithelial	231.79 (152.83 - 305.65)	275.09 (188.49 - 356.60)	213.96 (141.62 - 282.73)	0.382
CD163 Total	292.92 (201.86 - 396.59)	310.75 (221.60 - 472.74)	331.12 (219.05 - 349.46)	0.672
CD163 Epithelial	35.66 (16.56 - 61.13)	20.38 (5.09 - 68.78)	20.38 (12.74 - 58.08)	0.598
CD163 Subepithelial	245.54 (163.78 - 364.23)	290.37 (181.87 - 414.67)	308.71 (170.15 - 332.91)	0.641
CD80 Total	303.11 (133.72 - 458.48)	188.49 (50.94 - 453.38)	152.83 (86.60 - 369.33)	0.532
CD80 Epithelial	43.30 (10.19 - 81.51)	20.38 (2.55 - 96.79)	15.28 (7.64 - 30.57)	0.354
CD80 Subepithelial	213.96 (122.26 - 396.07)	183.40 (40.75 - 397.34)	122.26 (66.22 - 359.15)	0.395
CD163/CD68 Total	1.18 (0.76 - 1.74)	0.92 (0.85 - 1.32)	1.25 (0.78 - 1.53)	0.740
CD163/CD68 Epithelial	1.50 (0.57 - 3.00)	0.50 (0.29 - 1.17)	1.50 (0.51 - 2.68)	0.164
CD163/CD68 Subepithelial	1.25 (0.69 - 1.70)	0.92 (0.83 - 1.36)	1.20 (0.76 - 1.52)	0.922
CD80/CD68 Total	0.86 (0.75 - 1.74)	0.56 (0.26 - 1.28)	0.67 (0.23 - 1.14)	0.263
CD80/CD68 Epithelial	1.00 (0.38 - 2.29)	0.40 (0.06 - 1.67)	0.87 (0.17 - 2.25)	0.432
CD80/CD68 Subepithelial	0.93 (0.53 - 1.46)	0.63 (0.24 - 1.35)	0.66 (0.14 - 1.21)	0.238
CD80/CD163 Total	1.05 (0.59 - 1.43)	0.46 (0.30 - 1.22)	0.57 (0.23 - 0.89)	0.090
CD80/CD163 Epithelial	0.89 (0.33 - 1.67)	0.50 (0.13 - 1.88)	0.66 (0.19 - 1.13)	0.557
CD80/CD163 Subepithelial	1.05 (0.58 - 1.40)	0.47 (0.26 - 1.23)	0.62 (0.32 - 0.89)	0.240

Statistical differences between histological grades (G1, G2, and G3) were assessed using the Kruskal-Wallis test. Abbreviations: G1–G3, Histological Grade (Broders' classification)

Supplemental Table S4. Macrophage immunophenotype density (cell/mm2) and M ratios by IFQ and HRC scores and DOI in OSCC.

**Marker / Compartment**	**IFG**			**HRM**			**DOI**		
	**Low (Median / IQR)**	**High (Median / IQR)**	**p-value**	**Low (Median / IQR)**	**High (Median / IQR)**	**p-value**	**Low (Median / IQR)**	**High (Median / IQR)**	**p-value**
**CD68 Total**	262.35 (161.74-346.41)	275.09 (183.39-364.24)	0.883	264.90 (213.96-366.78)	168.11 (91.70-305.65)	**0.012***	331.12 (229.24-540.00)	257.26 (203.77-324.76)	0.146
**CD68 Epithelial**	33.12 (10.19-42.03)	40.75 (16.56-59.86)	0.386	35.66 (20.38-56.04)	15.28 (10.19-40.75)	0.132	40.75 (25.47-50.94)	35.66 (17.83-57.31)	0.611
**CD68 Subepithelial**	231.79 (152.83-305.65)	216.51 (157.92-332.40)	0.858	234.33 (178.30-341.31)	152.83 (81.51-275.09)	**0.007***	305.65 (203.77-468.67)	219.05 (168.11-282.73)	0.112
**CD163 Total**	272.54 (208.23-318.65)	349.46 (210.13-443.45)	0.369	310.75 (244.52-404.48)	198.67 (127.36-257.26)	**0.009***	300.56 (219.05-489.04)	305.15 (233.06-380.80)	0.842
**CD163 Epithelial**	20.38 (10.19-62.91)	35.66 (16.56-59.86)	0.732	20.38 (15.28-61.13)	40.75 (5.09-81.51)	0.802	20.38 (5.09-61.13)	28.02 (14.01-62.91)	0.492
**CD163 Subepithelial**	241.72 (175.49-291.13)	317.88 (164.03-374.68)	0.335	290.37 (193.58-373.91)	124.30 (76.41-252.16)	**0.007***	259.80 (203.77-424.86)	291.90 (175.49-351.37)	0.774
**CD80 Total**	163.02 (126.09-380.80)	272.55 (118.44-497.96)	0.365	188.49 (127.36-463.57)	137.54 (112.07-427.91)	0.504	188.49 (127.36-407.54)	264.90 (120.99-482.67)	0.674
**CD80 Epithelial**	30.57 (5.09-73.87)	20.38 (11.46-91.70)	0.883	20.38 (10.19-71.32)	15.28 (0.00-101.88)	0.686	45.85 (10.19-71.32)	20.38 (3.82-91.70)	0.707
**CD80 Subepithelial**	140.10 (100.61-345.14)	219.06 (106.98-450.83)	0.546	183.40 (122.26-407.53)	117.17 (101.88-336.21)	0.143	183.40 (81.51-361.69)	213.96 (120.99-447.01)	0.580
**CD163/CD68 Total**	0.96 (0.76-1.73)	1.26 (0.93-1.54)	0.832	1.18 (0.83-1.63)	1.16 (0.86-1.41)	0.862	0.92 (0.79-1.70)	1.25 (1.00-1.54)	0.340
**CD163/CD68 Epithelial**	1.00 (0.52-2.00)	0.67 (0.46-1.75)	0.757	1.00 (0.50-2.00)	0.58 (0.25-1.67)	0.499	1.50 (0.52-2.00)	0.67 (0.46-1.75)	0.363
**CD163/CD68 Subepithelial**	0.92 (0.69-1.70)	1.25 (1.00-1.54)	0.732	1.17 (0.79-1.55)	1.21 (0.77-1.55)	0.603	0.92 (0.69-1.70)	1.25 (1.00-1.54)	0.256
**CD80/CD68 Total**	0.81 (0.48-1.10)	0.97 (0.45-1.76)	0.757	0.87 (0.48-1.54)	1.08 (0.43-1.90)	0.773	0.81 (0.48-1.10)	0.97 (0.45-1.76)	0.112
**CD80/CD68 Epithelial**	0.52 (0.14-2.29)	1.50 (0.19-1.96)	0.961	0.62 (0.17-2.13)	0.83 (0.00-2.10)	1.000	0.52 (0.14-2.29)	1.50 (0.19-1.96)	0.412
**CD80/CD68 Subepithelial**	0.76 (0.43-1.13)	1.13 (0.48-1.76)	0.807	0.86 (0.39-1.33)	1.10 (0.48-1.85)	0.773	0.76 (0.43-1.13)	1.13 (0.48-1.76)	0.112
**CD80/CD163 Total**	0.73 (0.44-1.43)	0.96 (0.40-1.32)	0.660	0.87 (0.42-1.33)	0.80 (0.42-1.90)	0.773	0.73 (0.44-1.43)	0.96 (0.40-1.32)	0.550
**CD80/CD163 Epithelial**	0.50 (0.07-1.67)	1.13 (0.34-2.04)	0.946	0.90 (0.33-1.65)	0.56 (0.00-2.16)	0.390	0.50 (0.07-1.67)	1.13 (0.34-2.04)	0.109
**CD80/CD163 Subepithelial**	0.76 (0.35-1.27)	0.88 (0.40-1.45)	0.807	0.77 (0.36-1.37)	0.74 (0.43-2.28)	0.499	0.76 (0.35-1.27)	0.88 (0.40-1.45)	0.550

Statistical significance was assessed using the independent samples Mann-Whitney U test. Abbreviations: IFG, Infiltration/Growth pattern (Bryne's classification); HRM, Histological Risk Model (Brandwein-Gensler classification); DOI, Depth of Invasion;

Supplemental Table S5. Univariable Kaplan–Meier analysis of malignant transformation-free survival according to clinicopathological characteristics and macrophage markers.

Variable	N	MT events n (%)	MTFS (at 24 months) (%)	P-value[b]
Age (median cut-off)				0.857
< 62 years-old	10	2 (20.0%)	80%	
≥ 62 years-old	11	3 (27.3%)	80.8%	
Sex				0.616
Female	9	2 (22.2%)	87.5%	
Male	12	3 (25.0%)	75%	
Location				0.438
Tongue/Floor of mouth	3	0 (0.0%)	100%	
Others	18	5 (27.8%)	77.8%	
Dysplasia grade classification (WHO)				0.094
Mild	3	0 (0.0%)	100%	
Moderate	5	3 (60.0%)	60%	
Severe	1	1 (100.0%)	0%	
Binary risk classification				0.007
Low risk	15	1 (6.7%)	93.3%	
High risk	6	4 (66.7%)	50%	
CD68 Total (median cut-off)				0.949
< 142.6	16	4 (25.0%)	81.3%	
≥ 142.6	5	1 (20.0%)	75%	
CD68 Epithelial (median cut-off)				0.026
< 10.2	15	2 (13.3%)	93.3%	
≥ 10.2	6	3 (50.0%)	50%	
CD68 Subepithelial (median cut-off)				0.731
< 132.5	17	4 (23.5%)	82.4%	
≥ 132.5	4	1 (25.0%)	66.7%	
CD163 Total (median cut-off)				0.784
< 211.4	15	3 (20.0%)	79.4%	
≥ 211.4	6	2 (33.3%)	83.3%	
CD163 Epithelial (median cut-off)				0.449
< 10.2	17	3 (17.6%)	81.9%	
≥ 10.2	4	2 (50.0%)	75%	
CD163 Subepithelial (median cut-off)				0.227
< 182.4	13	2 (15.4%)	83.9%	
≥ 182.4	8	3 (37.5%)	75%	
CD80 Total (median cut-off)				0.069
< 127.4	11	1 (9.1%)	90.9%	
≥ 127.4	10	4 (40.0%)	67.5%	
CD80 Epithelial (median cut-off)				0.081
< 5.1	15	2 (13.3%)	86.7%	
≥ 5.1	6	3 (50.0%)	62.5%	
CD80 Subepithelial (median cut-off)				0.146
< 107.0	14	2 (14.3%)	85.1%	
≥ 107.0	7	3 (42.9%)	71.4%	

[a] Kaplan–Meier mean survival time), restricted to the longest observed time. Values are in months. [b] P-value from the Log-rank (Mantel–Cox) test; shown once per comparison. MT, malignant transformation; MTFS, malignant transformation-free survival.

Supplemental Table S6. Multivariable analysis of survivals on variables with significant effect in univariable analysis.

Oral Malignant Transformation-free Survival			
**Variable[a]**	***p-value***	**HR**	**95% CI**
Binary histological risk classification	0.046	9.40	1.04-85.35
CD68+ epithelial	0.098	6.82	0.7-66.49

HR, Hazard ratio; CI, confidence interval for HR. [a] Variables included in multivariable Cox regression analysis using enter method: Binary histological risk classification, high risk vs low risk (reference category); CD68+ epithelial, ≥ median vs < median (reference category).
